# Adsorption of Recombinant Human β-Defensin 2 and Two Mutants on Mesoporous Silica Nanoparticles and Its Effect against *Clavibacter michiganensis* subsp. *michiganensis*

**DOI:** 10.3390/nano11082144

**Published:** 2021-08-23

**Authors:** Gabriel Marcelino-Pérez, Roberto Ruiz-Medrano, Salvador Gallardo-Hernández, Beatriz Xoconostle-Cázares

**Affiliations:** 1 Programa de Doctorado en Nanociencias y Nanotecnología, Centro de Investigación y de Estudios Avanzados del Instituto Politécnico Nacional, Av. IPN 2508, Ciudad de México 07360, Mexico; gabriel.marcelino@cinvestav.mx; 2 Departamento de Biotecnología y Bioingeniería, Centro de Investigación y de Estudios Avanzados del Instituto Politécnico Nacional, Av. IPN 2508, Ciudad de México 07360, Mexico; rmedrano@cinvestav.mx; 3 Departamento de Física, Centro de Investigación y de Estudios Avanzados del Instituto Politécnico Nacional, Av. IPN 2508, Ciudad de México 07360, Mexico

**Keywords:** mesoporous silica nanoparticles, antimicrobial peptides, human β-defensin-2, peptide adsorption, peptide release

## Abstract

*Solanum lycopersicum* L. is affected among other pests and diseases, by the actinomycete *Clavibacter michiganensis* subsp. *michiganensis* (*Cmm*), causing important economic losses worldwide. Antimicrobial peptides (AMPs) are amphipathic cationic oligopeptides with which the development of pathogenic microorganisms has been inhibited. Therefore, in this study, we evaluate antimicrobial activity of mesoporous silica nanoparticles (MSN5.4) loaded with human β-defensin-2 (hβD2) and two mutants (TRX-hβD2-M and hβD2-M) against *Cmm*. hβD2, TRX-hβD2-M and hβD2-M presented a half-maximum inhibitory concentration (IC_50_) of 3.64, 1.56 and 6.17 μg/mL, respectively. MSNs had average particle sizes of 140 nm (SEM) and a tunable pore diameter of 4.8 up to 5.4 nm (BJH). AMPs were adsorbed more than 99% into MSN and a first release after 24 h was observed. The MSN loaded with the AMPs inhibited the growth of *Cmm* in solid and liquid media. It was also determined that MSNs protect AMPs from enzymatic degradation when the MSN/AMPs complexes were exposed to a pepsin treatment. An improved AMP performance was registered when it was adsorbed in the mesoporous matrix. The present study could expand the applications of MSNs loaded with AMPs as a biological control and provide new tools for the management of phytopathogenic microorganisms.

## 1. Introduction 

One of the most important crops worldwide is tomato, the fruit of *S. lycopersicum* L., of which just in 2019 production exceeded 180 million tons [1]. Depending on the growth stage and environmental conditions, this crop can be negatively affected by stress, pests and diseases that not only cause damage to the stem and fruit, but also to the root system of the plant [2,3]. *Cmm* is a Gram-positive bacterium distributed worldwide that causes bacterial tomato canker disease, which can cause large economic losses to farmers if not treated properly and in a timely manner [4]. The use of contaminated seeds and asymptomatic transplants causes the propagation of the disease, detection methods are inefficient at differentiating *Cmm* from non-pathogenic *Clavibacter* strains [5]. However, the possibility exists to make detection methods more efficient by merging emerging and mature technologies. For example, the detection of pathogenic microorganisms by means of antibodies functionalized to nanomaterials [6]. The infection occurs in seeds at early stages or young seedlings. When infection occurs in adult plants it could be asymptomatic, eventually producing marketable fruits [4,7,8]. Depending on the climatic conditions and geographical position, *Cmm* can survive inside contaminated seeds and plant residues for months or even years [9,10].

Currently the control of *Cmm* and other diseases are based mainly on application of chemical pesticides [7,11]. However, their indiscriminate use has shown side effects such as high-water quality requirements for irrigation, reduction of fruit quality, soil erosion, dangers to both human and environmental health and mainly the development of pathogen resistance [2,12]. Other eco-friendlier alternatives have been proposed for the control of *Cmm*, among which the application of inorganic nanomaterials with antimicrobial activity such as nanoparticles (NPs) of Ag, ZnO, Cu, etc., the biological control (competition between microorganisms), the introduction of resistant cultivars and the direct application of biomolecules with antimicrobial activity to cultivars [13,14,15]. Defensins are relatively short amphipathic cationic oligopeptides with variable length, sequence and structures that protect their host from a wide variety of bacteria, fungi and viruses [16]. They are usually composed of 5–100 amino acid residues, characterized by having 6–8 cysteine residues that link together to give rise to the formation of disulfide bridges [16,17,18,19]. According to the disposition of their disulfide bridges, defensins are subdivided into three different subfamilies: α-defensins, β-defensins and θ-defensins [17]. Specifically, hβD2 is composed of 41 amino acid residues and as the other β-defensins bears a 6-cysteine motif to be stabilized by three conserved disulfide bridges; hβD2 antimicrobial activity has been evaluated against different human pathogenic strains such as *Actinobacillus actinomycetemcomitans*, *Candida albicans* and *Pseudomona aeruginosa* [19,20]. The exact mechanism by which hβD2 exhibits antimicrobial activity is unknown, however, thanks to the resolution of its atomic structure by means of X-ray diffraction, it has been hypothesized that the uniform surface distribution of positively charged residues allow hβD2 to interfere with the cell walls of bacteria by means of electrostatic interactions with the polar head groups of the wall, such as lipopolysaccharides (Gram-negative bacteria) and teichoic acids (Gram-positive bacteria) [16,17,21,22,23,24]. Despite their mentioned great properties, these biomolecules exhibit different drawbacks such as susceptibility to proteases, the fact the their activity is reduced at high salt concentrations, changes in pH and temperature and at concentrations above 10 μg/mL tend to produce pro-inflammatory effects on humans [25]. An alternative that has been proposed to avoid the aforementioned effects is to encapsulate the AMPs [26].

The matrix of the nanocapsules can be constructed from proteins, polysaccharides and synthetic polymers [27]. Mesoporous silica-based materials have been gaining ground in recent years; structural characteristics such as uniform distribution of pore size, high specific surface area, high pore volume and tunable pore size (2–50 nm) make them excellent candidates for the encapsulation and delivery of biologically active molecules [28,29]. The main advantages of adsorbing biomolecules within a mesoporous matrix are their protection against adverse factors that cause their degradation and when they are released, they do so in a controlled manner at specific sites [30]. Different studies have shown that biomolecules that are released in a controlled and gradual manner have greater reactivity compared to those that are not found within a mesoporous matrix [31,32,33]. Thanks to this property, it has also been possible to expand the application of drugs or biomolecules that in high concentrations were considered toxic or produced cytotoxic effects (AMPs) [30]. In this context, the present work aimed to synthesize MSN, to encapsulate recombinant hβD2 and two mutants and to evaluate their antimicrobial activity against phytopathogenic bacteria *Cmm*.

## 2. Materials and Methods

### 2.1. Reagents

N,N-dimethylhexadecylamine (DHMA, ≥95%, Sigma Aldrich, Saint Louis, MO, USA), Pluronic F127 (EO106PO70EO106, Mw = 12600, Sigma Aldrich), cetyltrimelammonium bromide (CTAB, ≥95%, Sigma Aldrich), NaOH (Macron Fine Chemicals, Radnor, PA, USA), tetraethyl orthosilicate (TEOS, ≥99%, Sigma Aldrich), anhydrous glycerol (J.T. Baker, Radnor, PA, USA), glucose (≥99.5%, Sigma Aldrich), imidazole (99%, Sigma Aldrich), Tris base (Tris, ≥99.8%, J.T. Baker), KH_2_PO_4_ (≥95%, High Purity, Santiago de Querétaro, QRO, Mexico), K_2_HPO_4_ (≥98%, Sigma Aldrich), NaCl (≥99%, J.T. Baker), pepsin from porcine gastric mucosa (Sigma Aldrich), yeast extract (DIBICO, Cuautitlán Izcalli, EDOMÉX, Mexico) and casein peptone (DIBICO). For the synthesis of MSN, deionized water with a resistivity of 18.2 MΩ•cm was used. For all other experiments double distilled water was employed.

### 2.2. Plasmids

**Figure 1:**
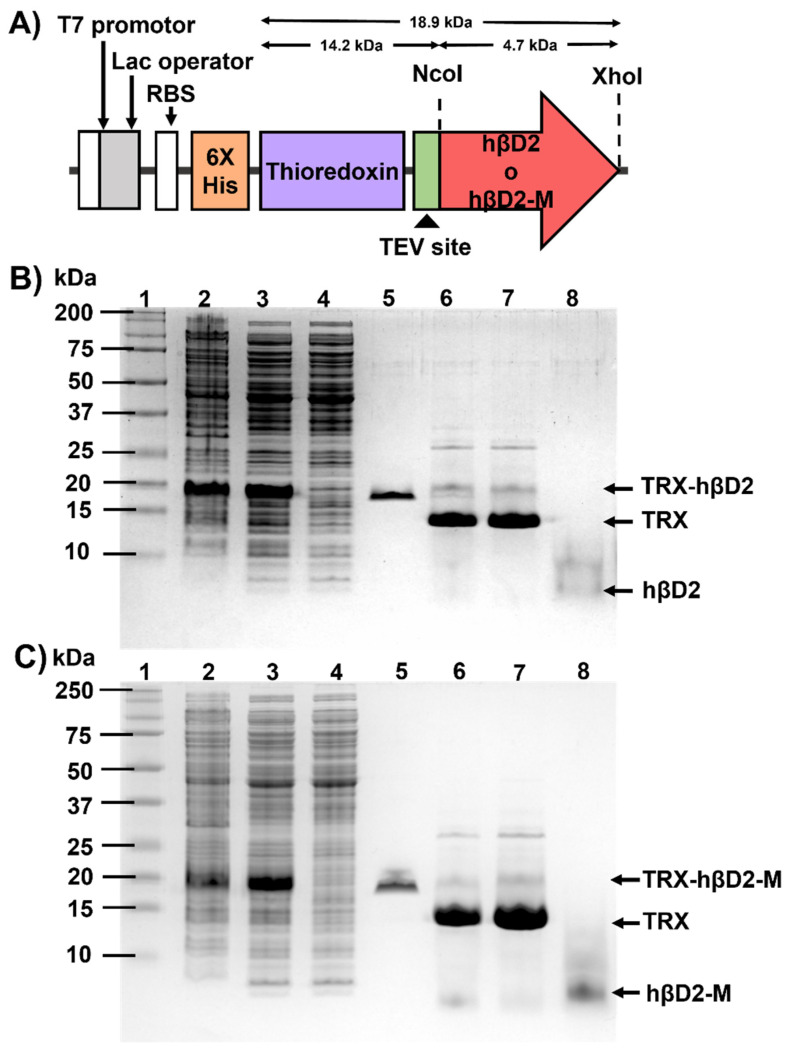
Construction, expression and purification of recombinant proteins. (**A**) Schematic representation of the reading frame of the expression vector TRX-hβD2 and TRX-hβD2-M, (**B**) expression and purification of hβD2; lane 1: molecular weight marker, lane 2: insoluble fraction, lane 3: soluble fraction, lane 4: fraction not bound to the first Ni^2+^-IMAC purification step, lane 5: fraction bound to the first Ni^2+^-IMAC purification step, lane 6: fraction after cleavage by TEV protease, lane 7: fraction bound to the second purification step by Ni^2+^-IMAC and lane 8: fraction not bound to the second purification step by Ni^2+^-IMAC. (**C**) expression and purification of hβD2-M; the charging plan is the same as for hβD2.

The amino acid sequence of hβD2 was obtained from the Protein Data Bank (1FD3). The sequence of the gene that codes for hβD2 with optimized codons for *Escherichia coli* was cloned between the restriction sites NcoI and XhoI within the vector pUC57 (pUC57-hβD2) by the company GenScript. The plasmid was reconstituted within Milli-Q water and transformed into chemocompetent *E*. *coli* Mach1 T1 cells. Subsequently, pUC57-hβD2 and the expression vector pCri-4a extracted with the commercial kit ZR Plasmid Miniprep-Classic (Zymo Research, Tustin, CA, USA) were subjected to a double restriction assay with the enzymes NcoI and XhoI. The restricted products hβD2 and pCri-4a were purified from with the Zymoclean Gel DNA Recovery Kit (Zymo Research) from a 1% agarose gel and ligated together with the enzyme T4 DNA Ligase. Chemically competent cells of *E*. *coli* Mach1 T1 were transformed with the pCri-4a-hβD2 construction. Recombinant plasmids were sequenced in both directions. The expression vector pCri-4a allows the expression of a translated fusion to a 6-histidine tag, followed by sequence encoding to the thioredoxin-binding protein (TrxA) and contain a tobacco etch virus protease (TEV) cleavage site (Figure 1A). In order to improve the intrinsic activity of hβD2 [34], a directed mutagenesis was carried out, incorporating a glycine residue at the amino terminal end and an arginine residue at the carboxyl end (TrxA-hβD2-M).

### 2.3. Expression of TRX-hβD2 and TrxA-hβD2-M

*E*. *coli* Rosetta (DE3) previously transformed with the plasmid pCri-4a-hβD2 or pCri--hβD2-M was seeded in 50 mL lysogeny broth (LB,) supplemented with kanamycin sulfate (50 μg/mL), chloramphenicol (20 μg/mL) and glucose (5 g/L). The culture was incubated at 37 °C for 16 h at 180 rpm for 16 h. 4 L of Terrific Broth medium (peptone 12 g/L, yeast extract 24 g/L, K_2_HPO_4_ 9.4 g/L, KH_2_PO_4_ g/L, glycerol 4 mL/L) supplemented with the same antibiotics and sugar mentioned above were inoculated with 1% from the preinoculum. The cultures were kept under a constant temperature (37 °C) and shaking (200 rpm) until the OD_600_ reached 0.7–0.8. The expression of the fusion proteins was induced with 1 mM isopropyl β-d-galactopyranoside (IPTG). After carrying out the induction, the culture was incubated at 18 °C for 16 h at 150 rpm. The cells were harvested by centrifugation at 5000× *g* for 30 min at 4 °C and stored at −80 °C until protein extraction.

### 2.4. Purification of TRX-hβD2 and TrxA-hβD2-M

Cell pellets (approximately 15 g) were resuspended in buffer A (20 mM NaH_2_PO_4_, 0.5 M NaCl, 20 mM imidazole, pH 7.4) and then lysed by sonication within an ice bath using the Ultrasonic Homogenizer 750 (Cole-Parmer, Vernon Hills, IL, USA). The cell lysate was centrifuged at 164,391× *g* for 30 min at 4 °C. The supernatant obtained loaded on a HisTrap HP 5 mL column previously equilibrated with buffer A. Proteins were eluted through a linear gradient (1–100%) with buffer B (NaH_2_PO_4_ 20 mM, 0.5 M NaCl, 500 mM imidazole, pH 7.4) for 15 CV, the fusion proteins were resolved in 10% Tricine-SDS-PAGE. To carry out the cleavage of the fusion proteins by TEV protease, the eluted fractions containing the protein of interest were subjected to a buffer exchange from B to C (1 mM reduced glutathione, 25 mM Tris-HCl, 150 mM NaCl, pH 7.4) using a HiPrep 26/10 Desalting column. The desalted fractions were mixed and the TEV enzyme was added in a ratio of 1:100 *v/v*, the reaction mixture was incubated at 4 °C for16 h. After cleavage process, the samples were re-purified using a HisTrap HP column using the previously mentioned conditions. The proteins that did not bound to the column, (corresponding to the AMPs) were concentrated to 0.5 mL with a centrifugal concentrator with a 3 kDa cut-off. To determine the concentration and yield of total protein in each of the purification processes, a commercial Pierce ™ BCA ™ kit (ThermoFisher Scientific, Rockford, IL, USA) was used.

### 2.5. Synthesis of Mesoporous Silica Nanoparticles (MSN)

The MSNs were synthesized based on the reported by Gu et al. [35], with the following modifications for increasing the pore diameter (Table S1). The synthesis of MSN carried out in this work is based on the self-assembly of negatively charged silicates (TEOS) and micelles composed of positively charged CTAB and DMHA under basic pH conditions, where F127 acts as a size inhibitor and dispersing agent to the same time by encapsulating the previous complex. The method is as follows: Treatment 1: at 22–25 °C 0.5 g CTAB (structure directing agent) and 50 mg of F127 were dissolved on 0.4 mL DHMA. Deionized water (240 mL) and 2 M NaOH (1.75 mL) were added to the mixture by vigorous stirring (600 rpm). After the solution becomes clarified, it was transferred to a flat-bottomed flask where the temperature was increased to 80 °C. Once the temperature was stabilized, 2.5 mL of TEOS was added dropwise and the flask was refluxed by maintaining the same temperature and stirring conditions for 2 h. At the end, the reaction mixture was cooled to room temperature and MSNs were concentrated and washed with ethanol once and water twice by centrifugation. To remove the surfactant template, 1 g of the material obtained was dissolved within an ethanol solution acidified with hydrochloric acid (1 mL of 37% HCl and 100 mL of absolute ethanol) and refluxed at 80 °C for 24 h to 600 rpm. The MSNs were concentrated and washed again by centrifugation with methanol twice. The material obtained was dried at 50 °C for 16 h and stored for further use. 

### 2.6. MSN Characterization

The particle size distribution of the NPs was determined by dynamic light scattering (DLS) with a Zetasizer Nano ZSP (Malvern Panalytical, Malvern, UK). To find out the possible functional groups of the material under study, Fourier transform infrared spectroscopy (FT-IR) was performed using an infrared spectrophotometer Nicolet 6700 (Thermo Electron Scientific Instruments Corporation, Madison, WI, USA). Raman spectroscopy was used to determine the different vibrational modes of the MSN, for this analysis the AFM-Raman NTEGRA SPECTRA II system (NT-MDT, Moscow, Russia) was used using a blue solid-state laser (455 nm). The analyses were carried out at room temperature by scanning a range of 200–1400 nm in the Raman shift. The morphology, size and porosity of the MSN were determined by transmission electron microscopy (TEM) and scanning (SEM). The samples for TEM were visualized in a JEM 2010 transmission electron microscope (JEOL, Peabody, MA, USA) using an acceleration of 200 KV. The samples were also observed in an AURIGA 3916-FESEM scanning electron microscope (CARL ZEISS, Jena, Germany) using an acceleration of 2 KV. The nitrogen adsorption-desorption isotherms of MSN were obtained using a Minisorb II instrument (BEL, Osaka, Japan) at 77 K. Prior to analysis, the samples were degassed at room temperature for 24 h. Specific surface areas were determined based on adsorption data using the Brunauer-Emmett-Teller (BET) model whilefrom the branches of the desorption isotherms and using the Barret-Joyner-Halenda (BJH) model, the effective volume and size distributions of pores were determined. Finally, the total pore volume was calculated based on the amount of N_2_ absorbed at a relative pressure (P/P_0_) of 0.99.

### 2.7. Antimicrobial Activity Assay and Determination of IC_50_

*Cmm* was cultivated in Mueller-Hinton broth (MHB) at 37 °C for 16 h at 180 rpm. Employing 0.5 mL of the previous culture, 50 mL of fresh MHB were inoculated. The culture was maintained under the conditions mentioned above until the OD_600_ reached 0.2–0.3. 9.5 mL of MHB agar prewarmed to 37 °C were added to 0.5 mL of the culture. It was homogenized and then poured onto a Petri dish containing MHB agar to form a uniform layer of 2 mm. Once the surface was dry, different volumes (5, 10 and 15 μL) of the defensins under study (50 μM) were added to the surface. The plates were incubated for 24 h at 28 °C, then the formed inhibition halos were measured. CH_3_COOH (acid acetic)10 mM pH 2.4 and Tris 50 mM pH 8.4 were used both as negative controls. The IC_50_ was determined based on that reported by Tay and Liu [36,37]. This measurement was defined as the minimum concentration of AMP that is required to inhibit 50% of the growth of *Cmm*.

### 2.8. Adsorption/Release of AMPs by MSN5.4

A microtube were filled with 5 mg of MSN5.4 (previously sterilized by UV light), 0.5 mg of AMPs and an amount of PBS 67 mM pH 7.0 to complete a final volume of 1 mL. The mixture was kept under constant stirring for 16 h at 4 °C. The samples were centrifuged at 10,000× *g* for 15 min at 4 °C at the end of the incubation period. The supernatant was carefully removed and analyzed by the Pierce ™ BCA Protein kit to determine the percentage of total protein concentration and thus indirectly estimate the adsorption of AMPs by MSN5.4. AMPs in PBS at the same concentration were used as controls.

About the release of AMPs, the methodology referred to in the previous paragraph was re-performed. The removal of the supernatant was done, and the pellet was dried in sterile conditions for 2 h. After 1 mL of sterilized PBS was added to the pellet and shaken with the aid of a vortex to obtain a homogeneous mixture, the microtubes remained at constant agitation and temperature 28 ± 2 °C throughout the whole analysis period plotted (2, 6, 24, 48, 72, 96, 120, 144, 168, 192 and 216 h). For each sampling point, microtubes were centrifuged at 10,000× *g* for 15 min at 4 °C, the resulting pellet was resuspended in 1 mL of sterile PBS. Supernatants were kept being analyzed by the Pierce™ BCA Protein kit (Thermo Fisher, Rockford, IL, USA) to determine the total protein concentration that was being released. These experiments were performed by triplicates.

### 2.9. Antimicrobial Activity of MSN/AMPs Complexes

The antimicrobial activity of the MSN/AMPs complexes was evaluated both in solid and liquid media. For the solid medium, the adsorption process was carried out as described in the previous section. The pellet was resuspended in PBS (1 mL). Afterwards, different volumes of the MSN/AMPs mixture were deposited onto Petri dishes with MHB medium and *Cmm* (see antimicrobial activity test section and IC_50_ determination). The plates were incubated for 24 h at 28 °C, the diameter of the inhibition halos was measured. MSN (5 mg/mL) and AMPs (0.5 mg/mL) were employed as controls. These experiments were performed by triplicates.

To evaluate the antimicrobial activity of the MSN/AMPs complexes at liquid medium, a *Cmm* preinoculum was first placed on MHB medium and incubated at 28 °C for 24 h at 180 rpm. Cells were inoculated to MHB medium in proportion 1:100 *v/v* and kept under the same conditions until the OD_600_ reached 0.5. From the previous culture, 200 µL were taken and added to 40 mL of fresh MHB medium to have a cell concentration of 2.5 × 10^6^ colony forming units per mL (CFU/mL); this was referred as the “diluted culture”. To set the adsorption process of the AMPs by MSN, after removing the supernatant and drying the MSN/AMPs, complexes were resuspended in 3 mL of diluted *Cmm* culture and incubated at 28 °C for 24 h at 180 rpm. After incubation period, serial dilutions were performed up to 1 × 10^6^ of which 100 µL were taken and boxes were planted with MHB medium. The plates were incubated at 28 °C for 72 h and growth colonies were counted, expressed as CFU/mL. Controls were supplemented with MSN (5 mg/mL) and AMPs (0.5 mg/mL) in PBS [33]. The experiments were carried out by duplicate.

### 2.10. MSN against Enzymatic Degradation of AMPs

After removing the supernatant and drying the MSN/AMP complexes, 200 µL of the pepsin (1 mg/mL) were added and incubated at 37 °C for 16 h under constant shaking. The microtubes were then centrifuged at 5000× *g* for 10 min at 4 °C. The resulting pellet was washed twice with deionized water and centrifuged at 5000× *g* for 10 min at 4 °C between each wash. MSN/AMPs complexes were resuspended in 3 mL of diluted *Cmm* culture and incubated at 28 °C for 24 h at 180 rpm. After the incubation period, serial dilutions were performed up to 1 × 10^6^ of which 100 µL were taken and Petri dishes were poured with 30 mL MHB medium. The plates were incubated at 28 °C for 72 h and the colonies were counted, bacterial growth was expressed as CFU/mL. Controls were prepared with MSN (5 mg/mL) in PBS and AMPs (1 mg/mL) treated with protease (1 mg/mL) in a 5:1 ratio (peptide:enzyme) [33]. The experiments for this assay were performed by duplicate.

### 2.11. Statistical Analysis

Data of measurements of the growth inhibition halos and the CFU/mL were presented as the mean and the bars represent the standard deviation. Data analysis was performed using one-way analysis of variance (ANOVA). The Tukey’s test was used to calculate *p*-values, *p*-values of ≤ 0.0028 were considered statistically significant.

## 3. Results

### 3.1. Construction, Expression and Purification of Recombinant Proteins

Two gene constructs were designed, encoding the defensin hβD2 and the mutant (hβD2-M), the open reading frame (ORF) encoding these peptides, cloned in the expression vectors are shown in Figure 1A. Orientation of DNA fragments encoding hβD2 and hβD2-M into the pCri-4a vector was assessed by digestion with the restriction enzymes NcoI and XhoI, as well as DNA sequencing, confirming no DNA changes were present in the ORFs.

Recombinant proteins were present in both soluble and insoluble fraction. The TRX-hβD2 fusion protein had a molecular weight of 18 kDa which coincides with the theoretical weight. After cleavage by TEV protease and analyzing the unbound fraction of a second purification step by Ni^2+^-IMAC, a band near to 4.6 kDa assigned to hβD2 was observed. Similar results were obtained for TRX-hβD2-M and hβD2-M, the complete purification processes for both peptides are summarized in Figure 1B,C. The yields of the hβD2, hβD2-M and TRX-hβD2-M proteins were 1.23, 1.38 and 11.96 mg per liter of culture respectively, with more than 90% purity according to a densitometry analysis.

### 3.2. Antimicrobial Activity Assays against Cmm

**Figure 2:**
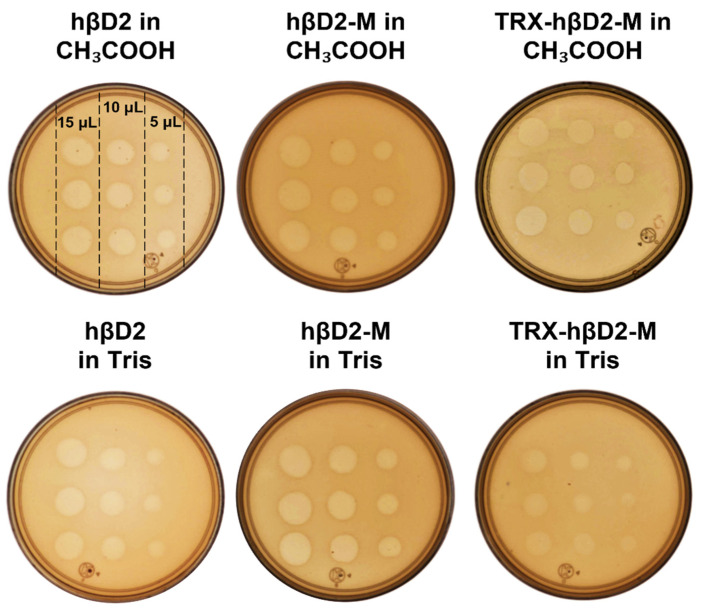
Antimicrobial activity of AMPs by the plaque diffusion method. Different volumes of the sample of interest were deposited on each plate in triplicate at a concentration of 50 µM.

The antimicrobial activity of AMPs under both basic and acidic pH conditions against *Cmm* was evaluated. CH_3_COOH pH 2.4 and Tris 50 mM pH 8.4 did not show antimicrobial activity against the strain under study, this same behavior was observed for TRX-hβD2 under both pH conditions. On the other hand, hβD2, TRX-hβD2-M and hβD2-M exhibited inhibition halos at the evaluated pH (Figure 2); for the three AMPs, the zones of inhibition increased as the sample volume increased.

The AMPs that inhibited the growth of *Cmm* on the plaque diffusion method, hβD2, TRX-hβD2-M and hβD2-M exhibited strong antimicrobial activity against *Cmm* with IC_50_ values between 1.5 and 20 µg/mL (Table 1). The activity of hβD2-M was higher than hβD2 and TRX-hβD2-M under both acidic and basic pH conditions.

### 3.3. MSN Characterization

**Figure 3:**
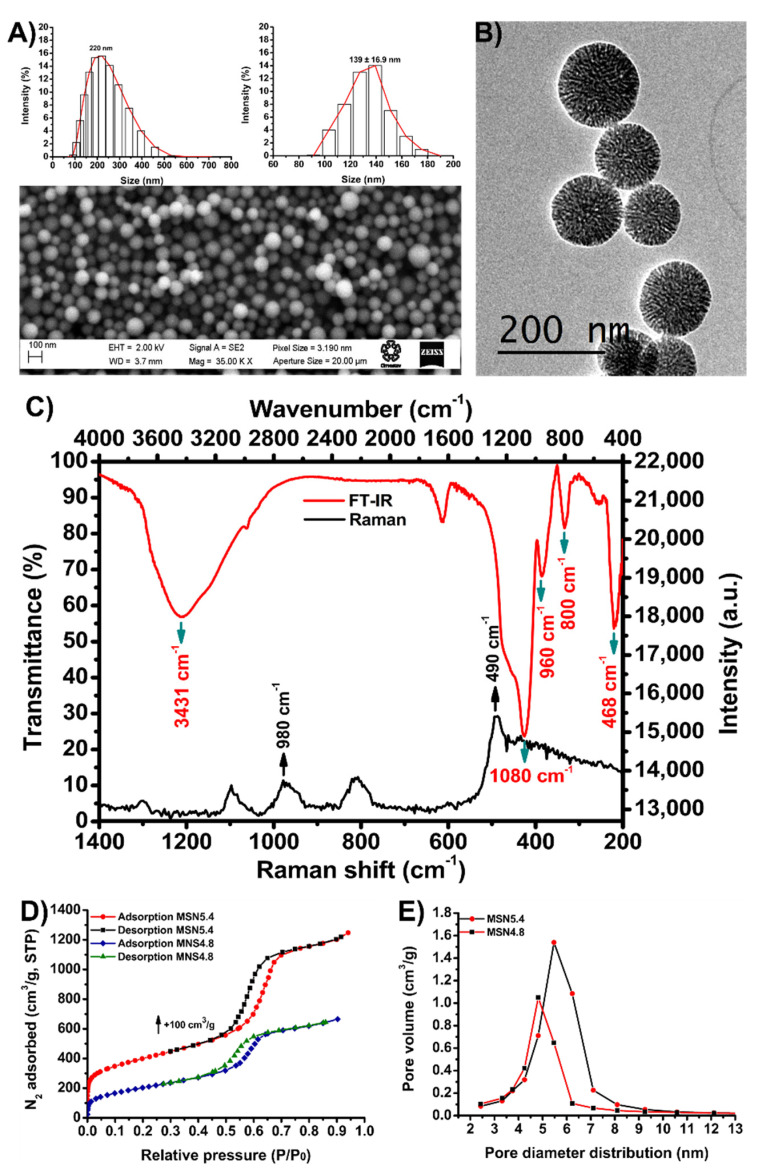
Characterization of MSN5.4. (**A**) SEM micrograph, (**B**) TEM micrograph, (**C**) FT-IR and Raman spectra, (**D**) nitrogen adsorption-desorption isotherms and (**E**) pore diameter distribution (BJH model).

Based on the different treatments that were carried out (Table S1), the conditions with which it is possible to obtain MSN with sizes lower than 150 nm were found. According to DLS analysis, it was determined that the particles obtained had a wide size distribution with a mode of 220 nm when resuspended in PBS (right inset of Figure 3A), this result was corroborated by SEM analysis where particles of spherical morphology with sizes ranging from about 80–140 nm were observed (Figure 3A and left insert of Figure 3A). By TEM it was possible to appreciate aggregated spherical particles of sizes like those obtained by SEM with disordered mesopores of variable size (Figure 3B). In order to determine the functional groups involved in the adsorption of AMPs, the material was analyzed by FT-IR (Figure 3C). The spectrum obtained presented vibration bands centered at 3431, 1080, 960, 800 and 468 cm^−1^ attributed to stretching vibrations of the O-H group of the water molecules linked to hydrogen (H-O-H-H), stretching vibrations of the Si-O covalent bond, stretching vibrations in the plane of the Si-O bond of the silanol groups (Si-OH), vibrations symmetric tension of the Si-O-Si bonds and symmetric bending vibrations of the Si-O-Si bonds of the siloxane bonds, respectively [38,39]. Moreover, the Raman spectrum obtained exhibited peaks centered at 490 and 980 cm^−1^ associated with the vibration of oxygen in Si–O–Si and the vibration of the silanol group (Si-O-H), respectively (Figure 3C). The relative amplitude of the last peak has been attributed to a high specific surface area [40]. It is important to mention that these results only refer to the material obtained by treatment 1.

The N_2_ adsorption-desorption results present isotherms type IV and hysteresis loops H1, where these patterns the presence of mesoporous analysis revealed the obtained material by treatment 1 (MSN5.4) had the best characteristics with mean pore size, pore volume (BJH model) and specific surface area (BET model) of 5.4 nm, 1.77 cm^3^/g and 1021.4 m^2^/g, respectively (Figure 3D,E). The results of these analyses are summarized in Table 2. According to the results, the modifications made to the methodology proposed by Gu [35] had a significant effect on the pore size of the MSN since it increased from 4.6 to 4.8 and 5.4 nm for the MSN-4.8 and MSN-5.4, respectively.

### 3.4. Adsorption and Release of AMPs

Based on the characterization results, MSN5.4 was chosen to carry out the adsorption and release tests of the AMPs under study. Regarding adsorption, 99.14, 99.08 and 99.46% of the evaluated concentration of hβD2, TRX-hβD2-M and hβD2-M were adsorbed on the MSN, respectively (Figure S1). This means that of the 500 mg of AMPs that were added, more than 495 mg were adsorbed in the MSN.

**Figure 4:**
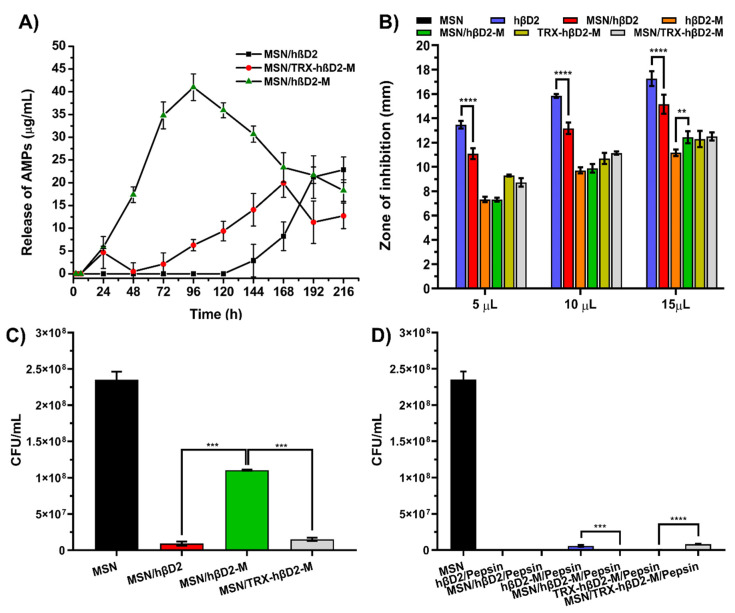
Characterization of the MSN/AMPs complexes. (**A**) Concentration of AMPs released from MSN5.4, (**B**) evaluation of antimicrobial activity by plaque diffusion (**, *p* ≤ 0.0028; ****, *p* < 0.0001), (**C**) evaluation of antimicrobial activity in liquid medium (***, *p* ≤ 0.0001) and (**D**) effect of pepsin on MSN/AMPs complexes (***, *p* ≤ 0.0005; ****, *p* < 0.0001).

Desorption assays, performed with hβD2-M and TRX-hβD2-M demonstrated the release of the proteins of 5 µg/mL at 24 h. hβD2-M and TRX-hβD2-M released a maximum of 41 µg/mL and 20 µg/mL at 96 and 168 h, respectively. On the other hand, hβD2 desorption was registered after 120 h (2.8 µg/mL); however, after that time its release rate was gradually increased, even exceeding TRX-hβD2-M (Figure 4A). As far as this experiment was monitored, it was determined that approximately 55, 81 and 229 µg of hβD2, TRX-hβD2-M and hβD2-M were released from MSN5.4, respectively (Figure S2).

### 3.5. Bioactivity of MSN Loaded with AMPs against Cmm

The bioactivity evaluation of the MSN5.4 loaded with the AMPs against *Cmm* was performed in both solid (qualitative) and liquid medium (quantitative). Figure 4B shows the bar graph corresponding to the measurements of the inhibition halos of MSN/AMPs. In general, the three AMPs adsorbed by MSN5.4 presented antimicrobial activity, which was proportional to the sample volume. In contrast, MSN/PBS control did not show cell growth inhibition. There was no statistically significant difference between TRX-hβD2-M and MSN/TRX-hβD2-M in the three evaluated sample volumes. On the other hand, free standing hβD2 showed greater inhibition than MNS/hβD2, likely due to a greater diffusivity of the peptide in the medium, since hβD2 is slowly released from MSN. Opposite case, it was observed with hβD2-M and the MNS/hβD2-M complex at 15 µL given that hβD2-M presented a greater inhibition halo than MSN/hβD2-M. 

On the other hand, the tests carried out in liquid medium showed *Cmm* growth inhibition by 96, 47 and 94% when hβD2, hβD2-M and TRX-hβD2-M were adsorbed on MSN5.4 (loaded with 0.5 mg of AMPs), respectively (Figure 4C). In the same way as in the previous test, MSN/PBS (control) did not show bacterial inhibition against *Cmm*. To note is that AMPs without MSN5.4 completely inhibited the growth of *Cmm*. 

### 3.6. Effect of Peptidase on AMPs and the MSN/AMPs Complex

To determine whether MSN protected AMPs from enzymatic degradation, a proteolytic degradation study was carried out using the peptidase pepsin. Considering the diversity of proteases in nature, further investigation will be required to evaluate other enzymes likely present in tomato crops. The results of this preliminary study indicate that AMPs without the mesoporous matrix are not susceptible to the catalytic activity of pepsin since hβD2, TRX-hβD2-M and hβD2-M inhibited the growth of *Cmm* by 99.98, 99.92 and 97.57%, respectively (Figure 4D). However, the MSN/hβD2 and MSN/hβD2-M complexes treated with the protease showed higher antimicrobial activity since they inhibited the growth of *Cmm* by 100 and 99.9%, respectively. On the other hand, TRX-hβD2-M treated with pepsin presented a higher antimicrobial activity than the MSN/TRX-hβD2-M complex (inhibited the growth of *Cmm* by 96.53%).

## 4. Discussion

It has been shown that hβD2, as well as other AMPs, can inhibit the growth of multidrug resistant strains [22,34]. In the present work, the recombinant production of hβD2, TRX-hβD2-M and hβD2-M was carried out to evaluate their activity. When analyzing the unbound fraction of the last purification step for hβD2 and hβD2-M by Tricine-SDS-PAGE, bands with a weight of 4.5 kDa corresponding with theoretical weight of defensins (Table S2). However, a band of 9 kDa was also observed for both cases. Previous studies have reported that hβD2 coexists as a mixture of aggregated peptides that are mostly dimers and their multimerization state depends on their concentration (the higher the concentration the bigger amount of formed homo-oligomers), so the 9 kDa band can be attributed to the hβD2 dimer [22,41]. The hβD2 dimer is formed by the interaction of the β1 chains of both monomers, resulting in the formation of a six-chain β sheet, however, this interaction is limited by two hydrogen bridges between His16 and the skeleton of Cys15. However, it also involves the hydroxyl groups of Tyr24, this dimer is mainly stabilized by van de Waals forces, with residues Pro5, Ala13, Ile14, Cys15, His16 and Pro17 responsible for most of these interactions [41].

hβD2, TRX-hβD2-M and hβD2-M showed antimicrobial activity on *Cmm* both in solid and liquid medium. Based on the bibliographic review that was carried out, the IC_50_ value presented by hβD2 and hβD2-M (acid buffer) is lower than that reported for inhibiting *Cmm* using AMPs [42,43,44].

It should be mentioned that the hβD2-M mutant presented a lower IC_50_ than hβD2, the reason why hβD2-M exhibits better antimicrobial activity against *Cmm* compared to hβD2 could be attributed to our replacement of Pro45 by an Arg at the carboxyl terminal end. By changing the content of Arg in the sequence of an AMP, a higher net positive charge was obtained due to a guanidinium functional group present in the replaced amino acid. Thus a promoting of long-range electrostatic attraction towards negatively charged bacterial membranes is done [45,46]. The mutation carried out served its purpose by improving the antimicrobial activity of hβD2 on *Cmm*. Likewise, it is important to emphasize that hβD2-M presents antimicrobial activity even when it is fused to TrxA (TRX-hβD2-M), until now there are few reports indicating that an AMP presents activity linked to a fusion protein [47,48].

MSN5.4 and MSN4.8 presented a type IV adsorption-desorption isotherm of N_2_ with an imminent type of HI hysteresis loop which are characteristic of mesopores in the form of independent thin cylindrical channels with a wide size distribution [28,49]. On the other hand, the increase in the amount of DMHA accompanied by the decrease in TEOS in the synthesis of MSN0.9 and MSN0.9’ led to a considerable decrease in pore diameter, pore volume and surface area, therefore both materials presented a characteristic behavior of microporous materials like a type I isotherm (<2 nm); this could be attributed to the fact that the TEOS in less quantity entered directly into the hydrophobic area of the CTAB micelles, preventing the DMHA from fulfilling its role as a mediator of the pore size, in addition, the excess DMHA is not transferred to the CTAB micelles if not to those of F127 resulting in obtaining microporous materials [35,50].

Using different complementary techniques, we determined that the pore size and diameter for MSN5.4 were 140 and 5.4 nm, respectively. On the other hand, according to the Pymol *software*, the dimensions of the AMPs are less than the pore diameter of the MSN5.4 (Figure S3 and Table S2), so that AMPs can easily enter in the pores of the MSN5.4. The adsorption of hβD2, TRX-hβD2 and hβD2-M by MSN 5.4 was 99.14, 99.08 and 99.46%. When analyzing MSN5.4 by FT-IR and Raman, silanol (hydrophilic) and siloxane (slightly hydrophobic) groups were identified. The adsorption process of biomolecules by mesoporous matrices such as MSN 5.4 is mainly governed by hydrophilic and hydrophobic interactions of the groups. silanol and siloxane and the regions with the same nature of the biomolecules, however, as the silanol groups are more exposed, it would be expected that the hydrophilic interactions predominate [29,39]. Despite having the atomic structure of hβD2, it is difficult to ensure that residues carry out the interaction with the internal surface of MSN5.4.

By evaluating the desorption of AMPs by MSN5.4 in PBS by quantifying total protein for 216 h, we determined that hβD2-M and TRX-hβD2-M are released until after 24 and 48 h, respectively. TRX-hβD2-M presented desorption at 24 h, however, the amount of protein decreased at 48 h, this may be due to protein physiosorbed on the surface and not in the pores of the MSN and therefore it was easily released during the first 24 h [35]. hβD2 began to be resorbed until after 120 h. The release of MSN AMPs at neutral pH is carried out by a diffusion process through the pores that is controlled by electrostatic interactions [29,51]. 

The MSN/hβD2 and MSN/TRX-hβD2 complexes in liquid medium greatly inhibited the growth of *Cmm.* However, with no statistically significant difference between them within the conditions of our experiment. On the other hand, the MSN/hβD2 complex only inhibited the growth of *Cmm* by 53%, this result is nor consistent with what was obtained in the desorption tests neither the evaluation of antimicrobial activity in solid medium. With respect to the desorption test, hβD2 was released faster than the MSN compared to the other AMPs. On the solid medium the MSN/hβD2 complex presented inhibition halos of greater diameter than hβD2 without the matrix (15 µL). The above suggests that the material in the liquid MHB presented some alterations on its surface such as the blocking of its pores by some compound in the medium as a consequence the complex had a lower antimicrobial activity [33,52]. As mentioned above, the AMPs without the mesoporous matrix completely inhibited the growth of *Cmm*, this is because the IC_50_ concentration was exceeded. It is possible that hβD2-M modifies its charge (more cationic) but since it does not have the matrix present and the most anionic is the wall of the bacteria, its activity is not affected antimicrobial.

The adsorption and posterior release of multiple AMPs has been carried out in different mesoporous matrices [29,32,38], in general it has been determined that the matrix confers protection to AMPs from multiple factors and enhances their antimicrobial activity when they are release, a clear example is that reported by Durak et al. [33] who adsorbed bactofencin A within two different mesoporous matrices and when facing these complexes (MSN/AMPs) to the activity of several proteases, they observed that bactofencin A, had a better activity antimicrobial against *Staphylococcus aureus* compared to the peptide without the mesoporous matrix. It is very important to mention that our AMPs were not so susceptible to pepsin degradation. Since they still significantly inhibited the growth of *Cmm*, after exposition to pepsin. However, when they were adsorbed on MSN5.4, their antimicrobial activity was significantly higher. On the other hand, the MSN/TRX-hβD2-M complex presented a lower antimicrobial activity in contrast to the peptide without the matrix, here it is possible that the pepsin has carried out some type of interaction with the material causing the clogging of the pores and being TRX-hβD2-M the largest protein had a lower degree of release and therefore the complex presented a lower antimicrobial activity against *Cmm* [52]. According to the above, AMPs with and without MSN could be applied in a liquid medium to disinfect seeds and young seedlings, as well as to combat *Cmm* when it is infecting *S*. *lycopersicum* plants in advanced stages of development and in this way its spread could be reduced.

## 5. Conclusions

In this work, we demonstrated that recombinant expressed AMPs hβD2, TRX-hβD2-M and hβD2-M in *E*. *coli* inhibit the growth of *Cmm* in both solid and liquid tests. The MSN/AMPs complexes (hβD2 and TRX-hβD2-M) in solution after being treated with a pepsin were shown to have better antimicrobial activity against *Cmm* compared to AMPs without the mesoporous matrix. Thus, AMPs and complex MSN/AMPs are excellent candidates for the potential management of bacterial tomato canker, as well as other diseases caused by the genus *Clavibacter*.

## Figures and Tables

**Table 1:** IC_50_ of the recombinant AMPs.

Protein	IC_50_ (µg/mL)
hβD2 in Tris	3.61 ± 0.47
hβD2 in CH_3_COOH	3.64 ± 0.33
hβD2-M in Tris	1.75 ± 0.13
hβD2-M in CH_3_COOH	1.56 ± 0.16
TRX-hβD2-M in Tris	19.99 ± 3.55
TRX-hβD2-M in CH_3_COOH	6.17 ± 0.08

**Table 2:** Summary of the properties of the synthesized mesoporous silica matrices.

Mesoporous Silicates	Pore Diameter ^a^ (nm)	Poro Volume (cm^3^/g)	BET Surface Area (m^2^/g)
**MSN5.4**	5.4	1.77	1021.4
**MSN4.8**	4.8	1.02	711.4
**MSN0.9**	0.9	0.31	653.2
**MSN0.9’**	0.9	0.30	688.5

^a^ Calculated using the BJH model from the desorption isotherms.

## Data Availability

Data is contained within the article or Supplementary Material.

## Supplementary Materials

The following are available online at https://www.mdpi.com/article/10.3390/nano11082144/s1, Figure S1. % AMPs loaded onto MSN5.4 after 16 h incubation to 4 °C. Figure S2. Cumulative release of AMPs with respect to time. Figure S3. AMPS measurements with the Pymol *software*. Table S1. Different treatments performed for the synthesis of MSN. Table S2. Physicochemical characteristics of AMPs.
