# Supplementary material for: Adsorption of Recombinant Human β-Defensin 2 and Two Mutants on Mesoporous Silica Nanoparticles and Its Effect against Clavibacter michiganensis subsp. michiganensis
Source: Nanomaterials (Basel). 2021 Aug 23;11(8):2144. doi: 10.3390/nano11082144 (PMC8400394; doi:10.3390/nano11082144)
Supplement: Supplementary file 1 [file nanomaterials-11-02144-s001.zip › nanomaterials-1323765-supplementary.pdf]

nanomaterials

MDPI

Supplementary Information

Adsorption of Recombinant Human β-Defensin 2 and Two Mutants on Mesoporous Silica Nanoparticles and Its Effect against *Clavibacter michiganensis* subsp. *michiganensis*

Gabriel Marcelino-Pérez [1], Roberto Ruiz-Medrano [2], Salvador Gallardo-Hernández [3,*] and Beatriz Xoconostle-Cázares [2,*]

[1] Programa de Doctorado en Nanociencias y Nanotecnología, Centro de Investigación y de Estudios Avanzados del Instituto Politécnico Nacional, Av. IPN 2508, Zacatenco 07360, Ciudad de México, Mexico; gabriel.marcelino@cinvestav.mx

[2] Departamento de Biotecnología y Bioingeniería, Centro de Investigación y de Estudios Avanzados del Instituto Politécnico Nacional, Av. IPN 2508, Zacatenco 07360, Ciudad de México, Mexico; rmedrano@cinvestav.mx

[3] Departamento de Física, Centro de Investigación y de Estudios Avanzados del Instituto Politécnico Nacional, Av. IPN 2508, Zacatenco 07360, Ciudad de México, Mexico

* Correspondence: sgallardo@fis.cinvestav.mx (S.G.-H.); bxoconos@cinvestav.mx (B.X.-C.)

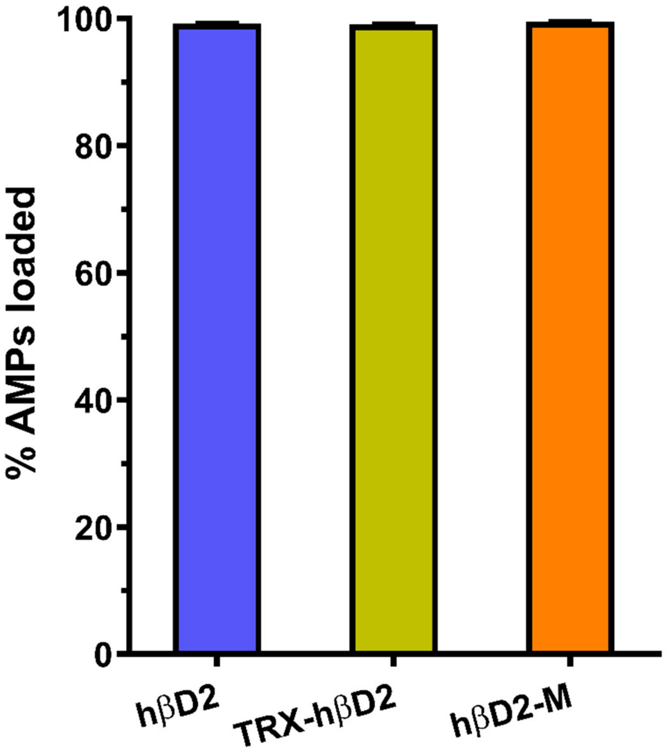

Figure S1. AMPs loaded onto MSN5.4 after 16 h incubation to 4 °C.

Table S1. Different treatments performed for the synthesis of MSN.

Reagents	Treatment 1 (MSN5.4)	Treatment 2 (MSN 4.8)	Treatment 3 (MSN0.9)	Treatment 4 (MSN0.9′)
F127 (O106PO70EO106)	50 mg	50 mg	50 mg	50 mg

DHMA (*N, N-dimethylhexadecylamine*)	0.4 mL	0.8 mL	0.4 mL	0.8 mL
CTAB (cetyltrimethylammonium bromide)	0.5 g	0.5 g	0.5 g	0.5 g
H_2O	240 mL	240 mL	240 mL	240 mL
NaOH (2 M)	1.75 mL	1.75 mL	1.75 mL	1.75 mL
TEOS (Tetraethyl orthosilicate)	2.5 mL	2.5 mL	1.1875 mL	1.1875 mL

Table S2. Physicochemical characteristics of AMPs.

**Protein**	**Sequence**	**Theoretical weight**	**Isoelectric pointa**	**Dimensions**
hβD2	GAMGIGDPVTC LKSGAICHPVF CPR-RYKQIGTCGLP GTKCCKKP	4.6 kDa	9.3	2.3 × 3.45 × 2.45 nm^3
TRX-hβD2-M	MKHHHHHHP MSDKIIHLTDDS FD TDVL-KADGAILVDF-WAEWCGPC KMIAPILDEI-ADEYQGKLT-VAKLNI DQNPGTAP-KYGIRGIPT-LLLFKNG EVAAT-KVGALSKGQLK EFLDANL AGSGSGSEN-LYFQGAMG-GIGDP VTCLKSGAICH PVFCPRRYKQIG TCGLPGTKCCK KR	18.9 kDa	8.1	3.97 × 7 × 3.08 nm^3
hβD2-M	GAMG-GIGDPVTCLKS GAICHPVF CPR-RYKQIGTCGLP GTKCCKKR	4.7 kDa	9.5	2.74 × 3.25 × 2.28 nm^3

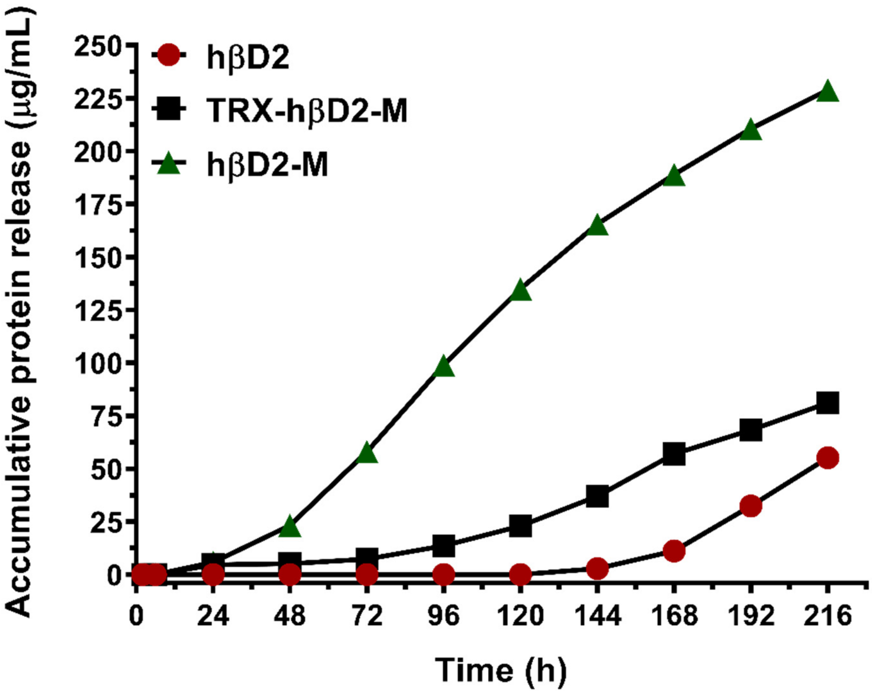

Figure S2. Cumulative release of AMPs with respect to time.

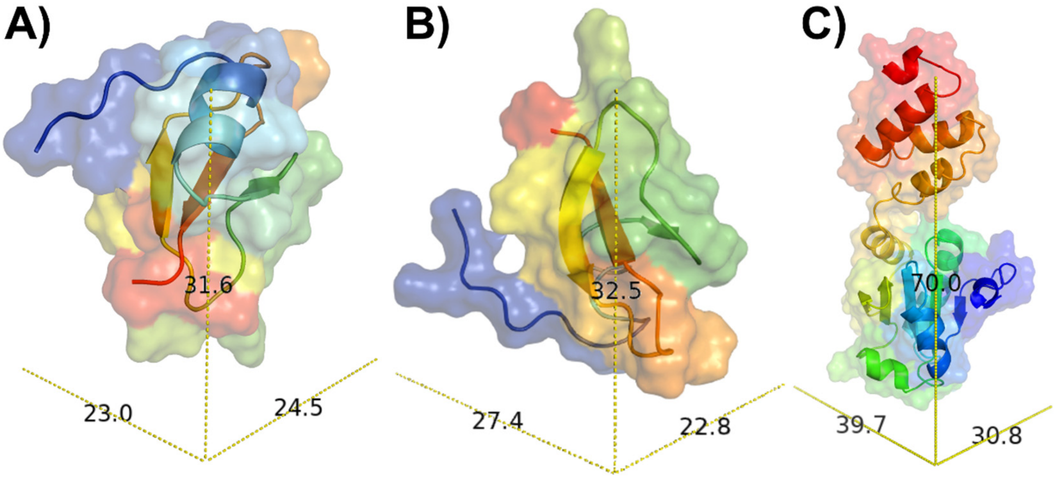

Figure S3. AMPS measurements with the Pymol software.
